# Iron-Modified Alkaline Lignin Chitosan Aerogel Microspheres for Sb(III) Removal in Water

**DOI:** 10.3390/molecules30204067

**Published:** 2025-10-13

**Authors:** Yaping Cheng, Haimin Liao, Huimei Shan, Yunquan Liu, Huinan Mo

**Affiliations:** 1 College of Environmental Science and Engineering, Guilin University of Technology, Guilin 541004, China; 2 Collaborative Innovation Center of Water Pollution Control and Water Security in Karst Area, Guilin University of Technology, Guilin 541004, China

**Keywords:** iron modification, alkaline lignin, chitosan, Sb(III) adsorption

## Abstract

Antimony (Sb), as a toxic heavy metal, has drawn worldwide attention, and its efficient removal from water has become increasingly urgent. In this study, an iron-modified alkaline lignin chitosan (Fe-ALCS) gel bead is prepared by the freeze-drying method to remove Sb(III) from the aqueous solution. The static adsorption experiment discusses the various environmental influences on the adsorption performance of Fe-ALCS for Sb(III) removal. The adsorption mechanism is explored by combining adsorption kinetics, isothermal adsorption, and characterization methods (such as FTIR, XRD, XPS, etc). The results show that the equilibrium adsorption capacity of Sb(III) decreases with the increase in pH and mass–volume ratio. With the increase in the initial Sb(III) concentration, *Q_e_* showed a rapid increasing trend in the range of 50–100 mg/L and continued to rise with the extension of contact time (*t*), reaching the maximum value at 3540 min. Under the optimal conditions of pH = 3, *m*/*v* = 1.0 g/L, and *C*_0_ = 20 mg/L, the removal efficiency (*R*_e_) value is 95.07%, which is still approximately 86.8% after five adsorption–desorption cycles. The maximum adsorption capacity is 266.58 mg/g fitted by the Langmuir model. The adsorption mechanism is mainly related to the iron-based active site of Fe–O(OH), where the O–H on its surface undergoes ligand exchange with Sb(OH)_3_ to form a stable Fe–O–Sb coordination structure. Additionally, C–OH, C–O, and other functional groups in ALCS also contribute to Sb adsorption. Fe-ALCS is an environmentally friendly, renewable, and convenient biomass adsorbent with good potential for wastewater treatment.

## 1. Introduction

With the rapid development of global urbanization and industrialization, the resulting wastes and wastewater have caused increasingly serious environmental pollution, especially water pollution [1,2,3,4]. Antimony (Sb) has been recognized as one of the harmful components in the water environment because of its high toxicity and wide distribution. Sb and its compounds (SbCl_3_, Sb_2_O_3_, Sb_2_O_5_, etc.) are widely used in the production of batteries, ammunition, pigments, and semiconductor components, as well as ceramic opacifiers and glass decolorizers. These account for a large proportion of global anthropogenic Sb emissions and pose a potential threat to ecosystems and human health [5,6]. It has been reported that Sb and its compounds easily enter the human body through skin contact, breathing, and the food chain, thus affecting human health [7,8,9,10]. In addition, compared with Sb(V) and other Sb-containing compounds, Sb(III) has received more attention from researchers due to its strong mobility and wide biotoxicity.

At present, there are many methods for removing pollutants from wastewater, such as coagulation/flocculation [11], ion exchange [12], membrane separation [13,14], bioremediation [15,16], and adsorption [17,18] etc. However, in practical applications, traditional treatment technologies, especially those centered on a single substance, such as bioremediation, precipitation, and membrane separation, often have obvious limitations. Not only are the treatment costs relatively high, but they may also cause secondary pollution and poor adsorption selectivity [19]. Based on this, more and more researchers choose to remove heavy metal ions from water by adsorption [17,18]. Meanwhile, developing a new type of composite material with high efficiency, environmental protection, and specific adsorption functions has become an urgent demand in the field of wastewater treatment.

Iron-based materials are considered to be the most promising and effective adsorbents because of their high surface activity, low cost, environmental benignness, and abundant reserves [20,21,22]. Chitosan (CS) is rich in amino and hydroxyl groups in its molecular structure, which have good adsorption and modifiable properties [23]. Additionally, lignin is a renewable biological macromolecule widely present in vascular plants. The global annual pulping production is approximately 70 million tons, but only 5% of vascular plants are utilized, with the rest mostly treated as waste [24,25,26]. Alkaline lignin (AL) is an important component of lignin, which comes from alkaline extraction of pulping black liquor and biomass. Its molecule contains many active functional groups, such as aliphatic hydroxyl groups, phenolic hydroxyl groups, carboxyl groups, and methoxy groups, which have ion exchange and adsorption. These advantages make alkali lignin a promising, environmentally benignness functional material [27,28,29,30,31,32]. Recently, researchers have continuously explored high-value utilization paths for lignin. For example, Albadarin et al. (2017) have prepared active lignin–chitosan extruded particles with a controllable particle size distribution to achieve efficient adsorption of methylene blue (MB), with a maximum adsorption capacity of 36.25 mg/g [33]. Sun et al. (2021) have prepared a novel lignosulfonate-modified graphene hydrogel (LS-GH) showing an ultra-high adsorption capacity of 1743.9 mg/g for Cr(VI) from wastewater [34]. Jong et al. (2021) have investigated the adsorption performance of arsenic (As) by zinc-modified lignin biochar (LBC), which is generated by the ZnCl_2_ colloid impregnation reaction and pyrolysis process, and the maximum adsorption capacity of arsenic is 20.2 mg/g [16]. The adsorption capacities of lignin for Cu(II) and Cd(II) are 87.05 and 137.14 mg/g, respectively. Masilompane et al. (2018) have synthesized a nano-adsorbent using sulfate lignin, chitosan, and TiO_2_ extracted from black liquor in papermaking and pulping [35]. They use nano-adsorbent to remove bright black dye from aqueous solutions, and the adsorption capacity of a single layer is 8.25 mg/g. Although the above studies have made good explorations in the removal of pollutants from composite materials prepared by modifying lignin–chitosan with metals (including various metals such as iron), they have rarely involved Sb(III) pollutants. Additionally, the preparation methods of such composite materials that have been reported are cumbersome and mostly rely on multi-step high-temperature reactions or special reagents [36,37].

This study aims to introduce iron (Fe) into the alkaline lignin (AL) and chitosan (CS) system to construct an aerogel microsphere structure. By regulating the valence state and morphology of iron to investigate the influence of iron on the adsorption performance and selectivity of Sb(III). The structural properties of the composite materials are analyzed using characterization techniques, including scanning electron microscopy (SEM), Fourier transform infrared spectroscopy (FTIR), powder X-ray diffraction (XRD), X-ray photoelectron spectroscopy (XPS), zeta potential, etc. Batch experiments are conducted to investigate the effects of factors (such as pH value, initial Sb(III) concentration, contact time, temperature, etc.) on the removal of Sb(III). Based on the characterization results, the acquired experimental data and kinetics and thermodynamics experiments reveal the adsorption mechanism of Fe-ALCS for Sb(III).

## 2. Results and Discussion

### 2.1. Effect of Fe Incorporating Amounts

**Figure 1:**
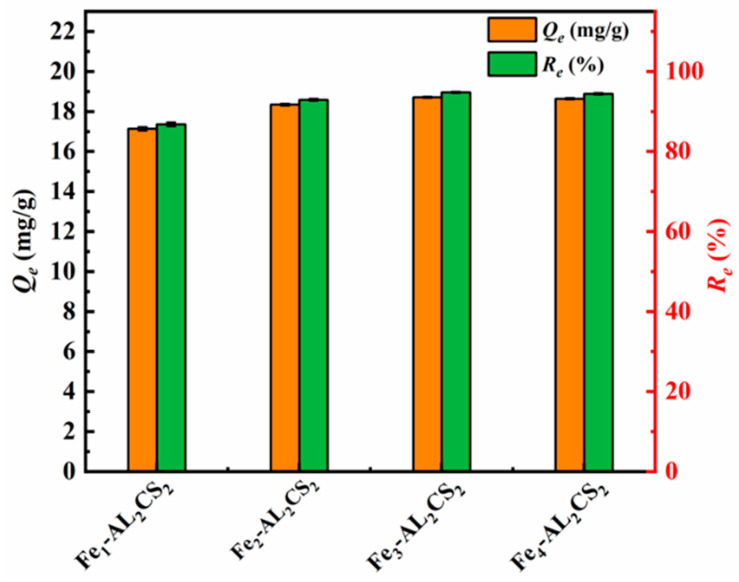
Variations in adsorption capacity (*Q_e_*) and removal efficiency (*R_e_*) at different mass Fe ratios for Sb(III).

The effects of Fe_n_-AL_2_CS_2_ (*n* = 1, 2, 3, 4) with different mass ratios on the adsorption of Sb(III) are studied, and the results are shown in Figure 1. The removal efficiency (*R_e_*) of Fe_1_-AL_2_CS_2_ for Sb(III) is only 86.79%, and the equilibrium adsorption capacity (*Q_e_*) is 17.13 mg/g. The *R_e_* value of Fe_2_-AL_2_CS_2_ and Fe_3_-AL_2_CS_2_ for Sb(III) are 92.92% and 94.80%, respectively, and their *Q_e_* values are 18.34 mg/g and 18.71 mg/g, respectively. This indicates that as the Fe content increases, the removal efficiency of Sb(III) by the material improves accordingly. Additionally, the *R*_e_ value of Fe_4_-AL_2_CS_2_ for Sb(III) is 94.43%, and the *Q_e_* value is 18.34 mg/g. Compared with Fe_3_-AL_2_CS_2_, the *Q_e_* and *R_e_* values decreased. This was attributed to the fact that as the Fe content increased, a high Fe content tended to transform from a highly dispersed state to an agglomerated state. Agglomerated Fe particles not only wrap some active sites, making it difficult for Sb(III) to come into contact, but also may clog the internal pores of the material and hinder the mass transfer and diffusion of Sb(III). Meanwhile, excessive Fe may fill or damage the pore structure of the AL_2_CS_2_ matrix, resulting in a reduction in the specific surface area of the material and a decrease in the effective contact area with Sb(III) [38]. In conclusion, Fe_3_-AL_2_CS_2_ has the best removal efficiency for Sb(III), and its spheroidizing property is superior to that of Fe_4_-AL_2_CS_2_. Therefore, Fe_3_-AL_2_CS_2_ is selected for the subsequent experiments.

### 2.2. Characterization

#### 2.2.1. VSM Analysis

**Figure 2:**
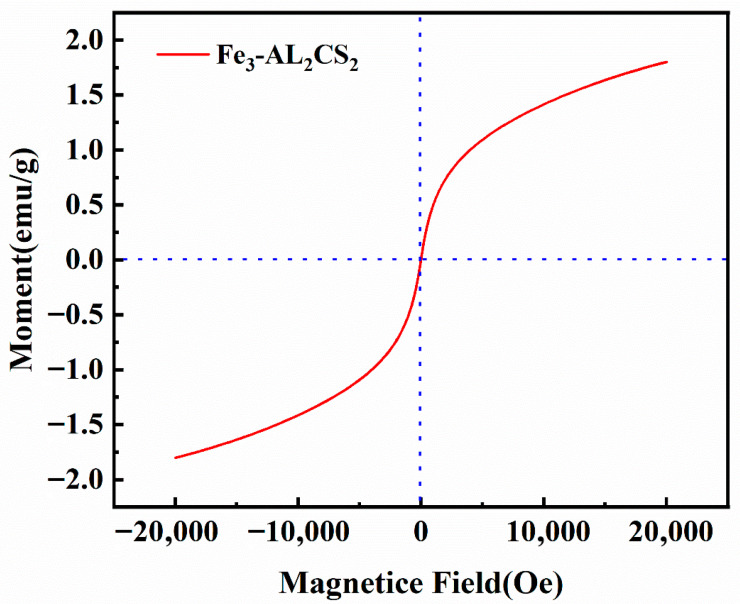
Magnetization curve of Fe3-AL2CS2.

Figure 2 shows the hysteresis curves of Fe_3_-AL_2_CS_2_ composite microspheres measured by a vibration sampling magnetometer (VSM). It can be seen that the hysteresis curve of Fe_3_-AL_2_CS_2_ has no hysteresis, and both the residual magnetism and coercive force are zero. This indicates that Fe_3_-AL_2_CS_2_ is superparamagnetized. In addition, the saturation magnetization of Fe_3_-AL_2_CS_2_ composite microspheres is 1.80 emu/g, which means that Fe_3_-AL_2_CS_2_ composite microspheres are difficult to separate from aqueous solutions using an external magnetic field.

#### 2.2.2. BET Analysis

**Figure 3:**
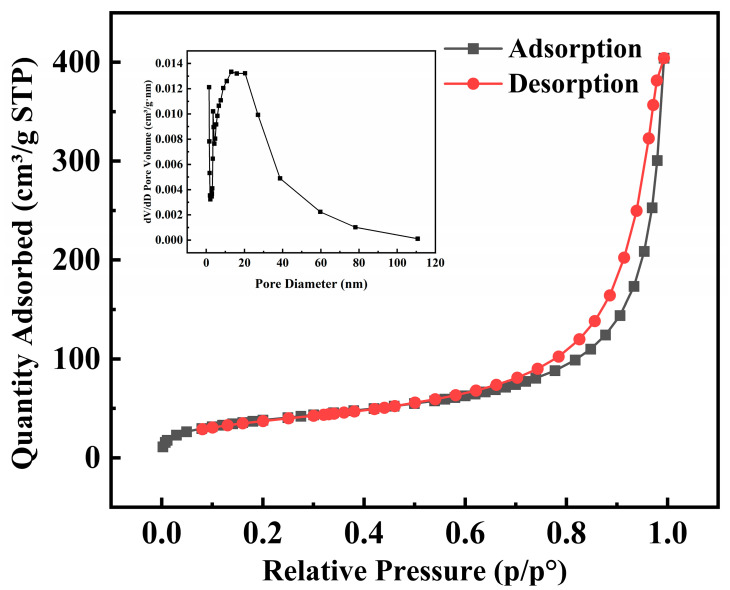
BET analysis of Fe_3_-AL_2_CS_2_.

The adsorption–desorption isotherms and pore size distribution of Fe_3_-AL_2_CS_2_ under N_2_ conditions are shown in Figure 3. The results show that the Fe_3_-AL_2_CS_2_ samples exhibit Type IV adsorption isotherms and show monolayer adsorption behavior accompanied by capillary condensation, and the specific surface area is 149.47 m^2^/g, with an average pore size of 18.09 nm. In addition, the hysteresis loop is overall narrow and has a low closure point. The gap between the adsorption branch and the desorption branch is small, forming a narrow loop, which belongs to the H4 tag hysteresis loop (IUPAC classification) [39]. Meanwhile, it can be known from the illustration that the pore volume is concentrated in the range of 2 to 50 nm, indicating that the material is mainly mesoporous [40].

#### 2.2.3. XRD and FTIR Analysis

**Figure 4:**
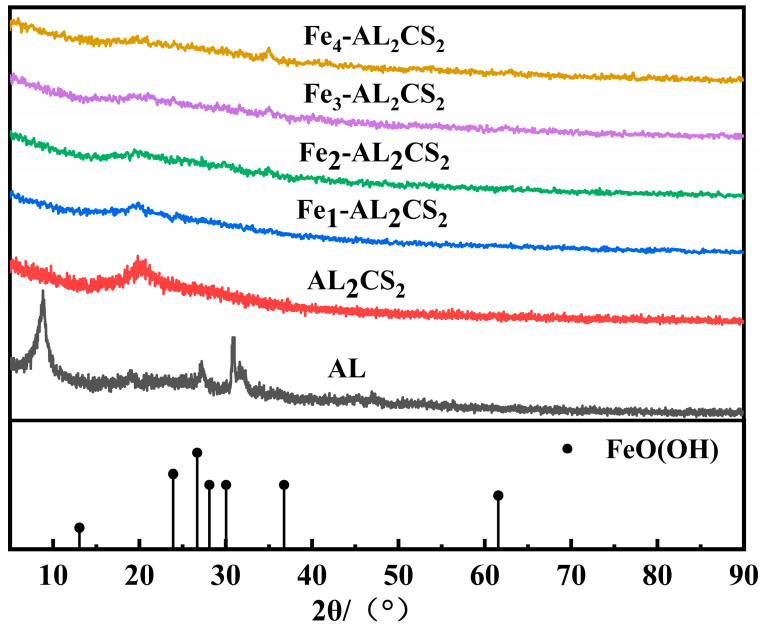
XRD analysis of AL, AL_2_CS_2_, Fe_1_-AL_2_CS_2_, Fe_2_-AL_2_CS_2_, Fe_3_-AL_2_CS_2_, and Fe_4_-AL_2_CS_2_.

Figure 4 shows the XRD patterns of the materials. As the amount of Fe gradually increases, distinct characteristic peaks are displayed at 2*θ* = 35°, indicating the formation of the crystal structure. Smaller wide peaks are shown at 2*θ* = 20.6° and 24.1°, similar to the characteristic peaks of standardized FeO(OH) (PDF No. 22-0353).

**Figure 5:**
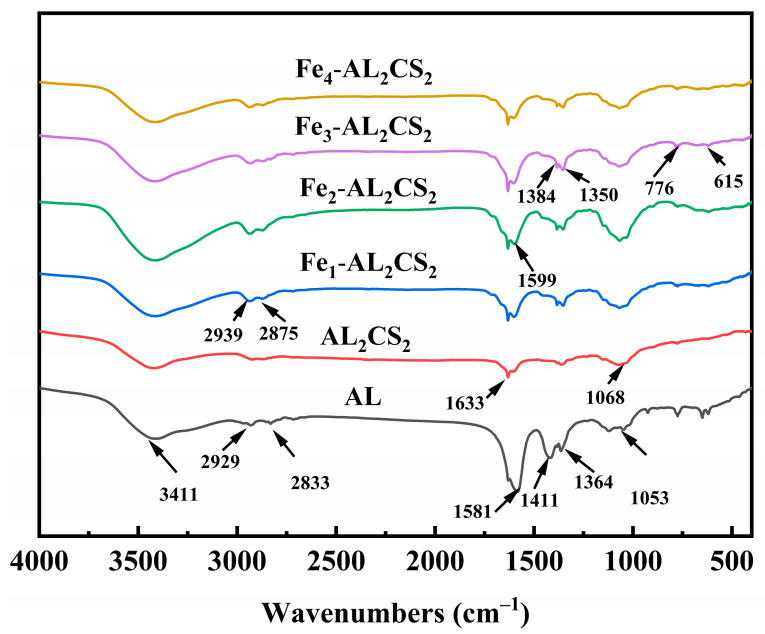
FTIR analysis of AL, AL_2_CS_2_, Fe_1_-AL_2_CS_2_, Fe_2_-AL_2_CS_2_, Fe_3_-AL_2_CS_2_, and Fe_4_-AL_2_CS_2_.

The Fourier transform infrared spectroscopy (FTIR) results are shown in Figure 5. It shows that all samples exhibit a wide O–H hydroxyl vibration peak at 3411 cm^−1^, corresponding to the –OH groups in AL and CS. The stretching vibrations of 2929 cm^−1^ and 2862 cm^−1^ correspond to the stretching of C–H bonds in the presence of aromatic, methoxy, and alkyl groups of AL, the vibration of 1581 cm^−1^ corresponds to aromatic C=C, and there is bending of O–H in the phenolic groups of 1411 cm^−1^ and 1364 cm^−1^. The 1053 cm^−1^ band corresponds to the C–O–C stretching vibration. Observation of the AL_2_CS_2_ spectrum reveals that with the introduction of CS, the peaks of 2929 cm^−1^ and 2862 cm^−1^ at AL basically disappear, with C=O corresponding to 1633 cm^−1^ and 1599 cm^−1^, and the peak of AL at 1581 cm^−1^ basically disappears. At 1053 cm^−1^, it is offset to 1068 cm^−1^, and this might be because the alkyl group of CS replaced the ether of the alkaline lignin. All of these indicate that alkaline lignin and chitosan have been successfully combined.

In the Fe_n_-AL_2_CS_2_ series of the spectral graph, it shifts from 2929 cm^−1^ and 2862 cm^−1^ relative to AL to 2939 cm^−1^ and 2875 cm^−1^. Significant intensity changes occurred at the peaks of 1633 cm^−1^ and 1599 cm^−1^. All these indicate that groups such as –OH, –OCH_3_, and C=O are involved in the modification of basic lignin chitosan by FeCl_2_. In addition, the peak at 776 cm^−1^ intensifies with the increase in Fe content, and a small new peak also appears at 615 cm^−1^, which is attributed to the stretching vibration of the Fe–O band. All these observations indicate the successful binding of FeO(OH) to ALCS.

#### 2.2.4. XPS Analysis

**Figure 6:**
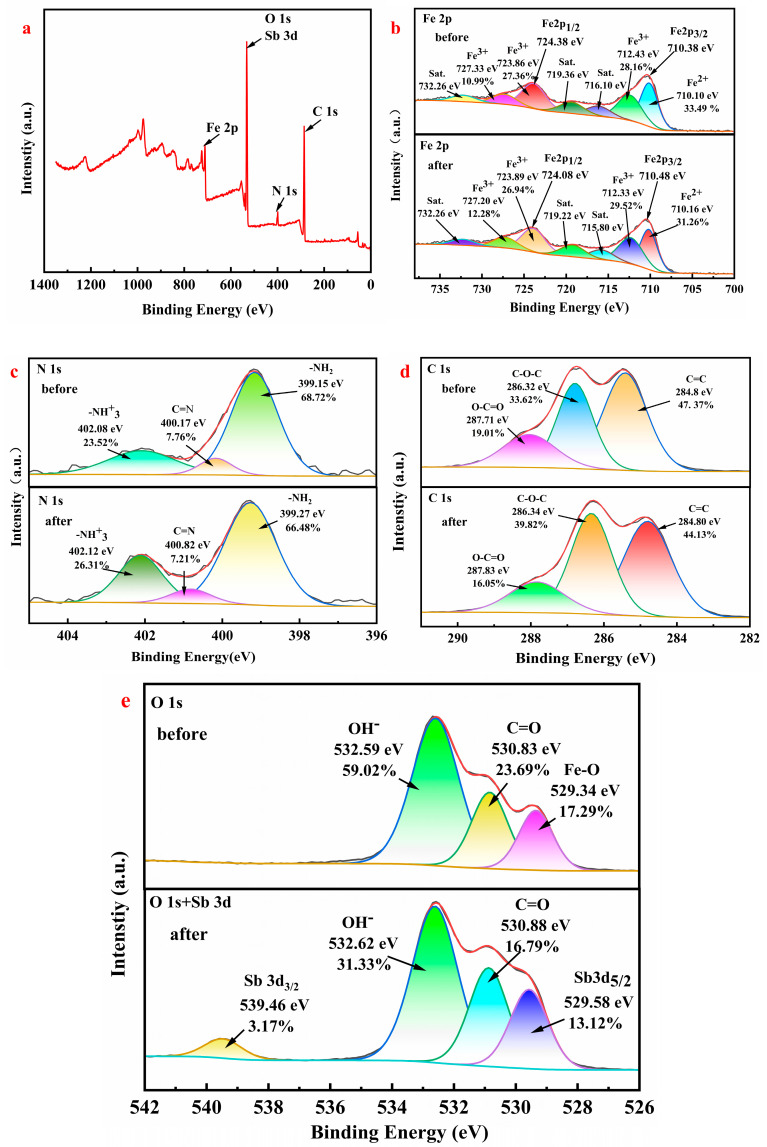
The full spectrum of XPS (**a**), the fine spectra of Fe 2p before and after adsorption (**b**), the fine spectra of N 1s (**c**), the fine spectra of C 1s (**d**), and the fine spectra of O 1s (**e**).

Figure 6a–e shows the X-ray photoelectron spectroscopy (XPS) spectrum. Figure 6a shows five distinct peaks, which are characteristic peaks of N, C, O, Sb, and Fe. The presence of these peaks confirms the adsorption of Sb(III) by Fe_3_-AL_2_CS_2_. The binding energy peaks observed in Figure 6b are 724.38 eV and 710.38 eV, respectively, which are assigned to Fe 2p_1/2_ and Fe 2p_3/2_, respectively. The high-resolution Fe2p spectra collected before adsorption show peaks at 710.110 eV and 712.43 eV, which are assigned to Fe^2+^ and Fe^3+^ species. Their contents are 33.49% and 28.16%, respectively. The spectrum also shows that the peaks of the two satellites are 716.10 eV and 719.35 eV, respectively. In Figure 6c, three peaks are observed in the N 1s spectrum before adsorption, at 399.15, 400.17, and 402.08 eV, which are –NH_2_, C=N, and –NH_3_^+^, respectively, and their contents are 68.72%, 7.76%, and 23.52%, respectively. The –NH_3_^+^ that appears at 402.08 eV is due to the reaction between the –NH_2_ of the original chitosan and the H^+^ of glacial acetic acid. After adsorption, the peaks corresponding to –NH_2_ and C=N both shifted to higher binding energies. Their binding energies increased by 0.65 eV and 0.12 eV, respectively, while their contents decreased by 0.45% and 2.24%, respectively. This indicates that chelation reactions occurred during the adsorption process. In comparison, the binding energy of –NH_3_^+^ increased by 0.04 eV, and its content also rose by 2.79%. This might be because –NH_2_ readily combines with protons (H^+^), undergoing protonation reactions, which leads to a decrease in –NH_2_ content and an increase in –NH_3_^+^ content [41]. In Figure 6d, there are three peaks within the binding energy range of 280.00–298.00 eV before adsorption, namely, 284.8, 286.32, and 287.71 eV. These peaks belong, respectively, to C=C (47.31%), C–O–C (33.62%), and O–C=O (47.37%). Moreover, after their contents are adsorbed, the peak values shift towards higher binding energies (C=C at 284.80 eV, C–O–C at 286.34 eV, and C=O at 287.83 eV), which might be a partial electron transfer between the synthetic material Fe_3_-AL_2_CS_2_ and heavy metal particles [42]. After adsorption, the binding energies of the C–O–C and O–C=O peaks increased by 0.02 eV and 0.12 eV, respectively. This change originated from the fact that the Sb(III) empty orbitals accepted the lone pair of electrons of the O atom through coordination. After the electron density of the O atom decreased, the electron cloud connected to C shifted towards O, increasing the binding energy. Among them, carboxyl oxygen has a more significant electron transfer with Sb(III), while the ether oxygen interaction is relatively weak. Moreover, the spectral peaks have not broadened, or new peaks have appeared, indicating that no chemical bond breakage has occurred, and it is an electron rearrangement. Consistent with the studies of Wang et al. [43] and Li et al. [44], both jointly verified that the mechanism of Sb(III) induced electron transfer of oxygen-containing functional groups through XPS and DFT calculations. In Figure 6e, in the O1s spectrum before adsorption, three peaks appeared at 526–540 eV, 529.34 eV, 530.83 eV, and 532.59 eV, respectively. They are Fe–O, C=O, and OH^−^, with contents of 17.29%, 23.69%, and 59.02%, respectively. Two characteristic peaks are observed in the O1s and Sb3d spectra after adsorption, corresponding to Sb3d_3/2_ (539.46 eV) and Sb3d_5/2_ (529.58 eV), respectively, with relative contents of 3.17% and 13.12%. This indicates that the material adsorbs Sb. It should be noted that the scanning area of Sb3d_5/2_ overlaps with that of O1s, which is attributed to the redox reaction between FeO(OH) and Sb(III). In FeO(OH), Fe^3+^ acts as an electron acceptor, capturing electrons from the outer orbital of Sb(III), causing Sb(III) to lose electrons and undergo oxidation, with its valence state rising from +3 to +5 and converting to Sb(V). At the same time, Fe^3+^ is reduced to Fe^2+^ due to the acquisition of electrons. This process is the fundamental cause of the formation of the Sb3d_5/2_ peak. This further confirms the adsorption and transformation mechanism of Sb by the material [43,45]. After adsorption, the proportion of OH^−^ significantly decreased, which is attributed to the cleavage of hydroxyl bonds in the composite material. This is due to precipitation or the formation of complexes with Sb(III) ions, which is consistent with previous reports [46,47,48].

### 2.3. Influencing Factors

#### 2.3.1. Effect of pH Value

**Figure 7:**
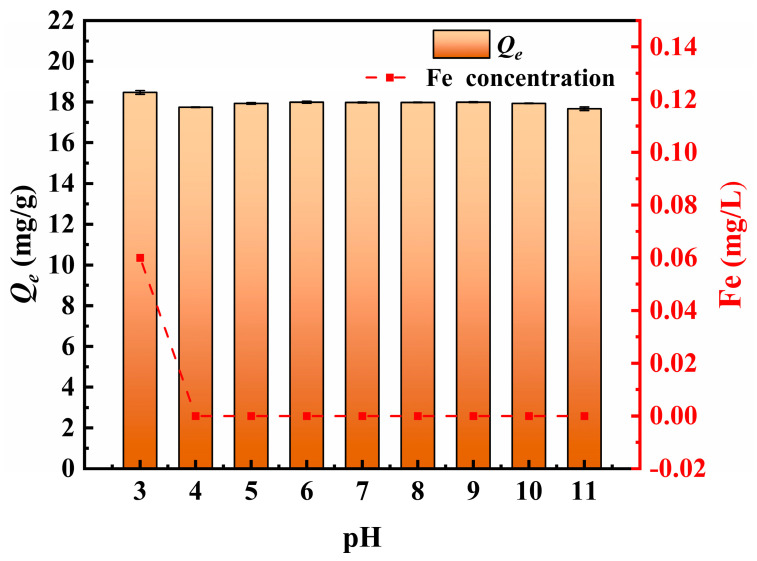
The changes in the adsorption capacity (*Q_e_*) of Sb(III) and the concentration.

The pH value not only alters the existence form of Sb(III) in water but also may affect the existence state of metal elements in materials [49]. As can be seen from Figure 7, under the condition of pH value ranging from 3 to 11, the equilibrium adsorption capacity (*Q_e_*) of Fe-ALCS for Sb(III) varies significantly with the pH value. When the pH value rose from 3 to 11, *Q_e_* decreased from 18.47 mg/g to 17.67 mg/g. Among them, the maximum dissolution amount of Fe was 0.06 mg/L when pH = 3. The dissolution amounts of Fe under other pH conditions were all 0, and all the dissolution amounts were lower than the drinking water Fe limit (0.3 mg/L) stipulated by the World Health Organization (WHO). It can be seen that Fe_3_-AL_2_CS_2_ has good safety and environmental compatibility. Moreover, as the pH value increases, *Q_e_* gradually decreases, which also indicates that its adsorption of Sb(III) is more efficient in acidic environments. Specifically, when the pH value is within the range of 3 to 10, Sb(III) mainly exists as neutral molecules HSbO_2_ and Sb(OH)_3_. Previous studies have confirmed that under acidic conditions, Sb (III) can adhere to the material surface by interacting with the ligands of the active functional groups on the adsorbent surface [50]. Moreover, at lower pH values, the protonation degree of the surface active functional groups of the adsorbent increases, and the number of effective active sites that can bind to Sb(III) increases, thereby enhancing the adsorption capacity [51]. However, as the pH value rises (under weakly acidic and alkaline conditions), the protonation process of the functional groups is inhibited, the number of effective binding sites decreases, and the adsorption effect weakens. The removal rate decreased accordingly. However, it should be noted that the removal rate is relatively higher when the pH is between 6 and 9 compared to when the pH is 4. This situation can be explained by the amphoteric metal property of antimony oxides. Within this pH range, as the concentration of OH^−^ increases, antimony is prone to combine with OH^−^ to undergo a precipitation reaction, generating Sb(OH)_3_ that is insoluble in water. This precipitation effect further reduces the content of free Sb(III) in water, complementing the removal effect of the adsorption process, and it ultimately slightly increases the overall removal rate [52]. In conclusion, based on the experimental results and the analysis of the adsorption mechanism, it is ultimately determined that pH = 3 is the optimal pH condition for subsequent experiments.

#### 2.3.2. Effect of the Mass–Volume Ratio

**Figure 8:**
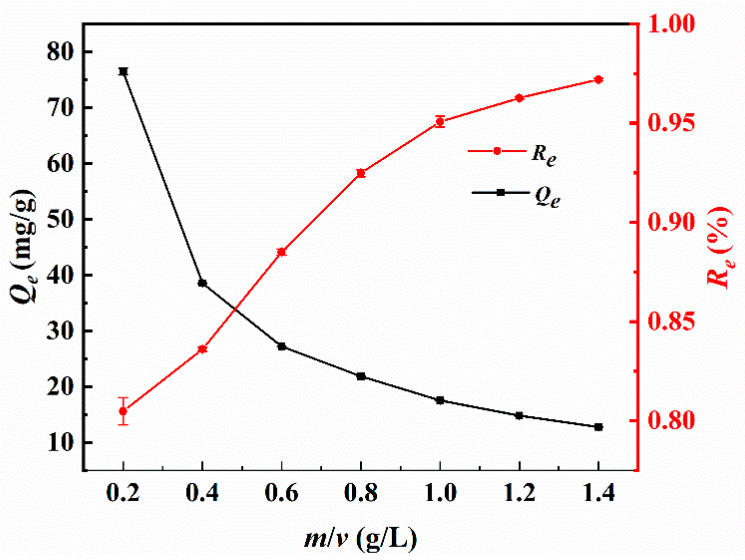
Changes in Sb(III) adsorption capacities (*Q_e_*) and removal efficiency (*R_e_*) with *m*/*v.*

To investigate the effect of mass-to-volume ratio (*m*/*v*) on the adsorption of Sb(III) by Fe_3_-AL_2_CS_2_, the *m*/*v* values of Fe_3_-AL_2_CS_2_ to Sb(III) solution are set at 0.2, 0.4, 0.6, 0.8, 1.0, 1.2, and 1.4 g/L for static adsorption experiments. The result is shown in Figure 8. It shows that with the increase in *m*/*v* value, the removal efficiency (*R_e_*) increases from 80.48% to 97.20%, while the equilibrium adsorption capacity (*Q_e_*) decreases from 76.50 mg/g to 12.79 mg/g. The increase in the *m/v* value refers to the increase in the dose of Fe_3_-AL_2_CS_2_, while the initial Sb(III) concentration remains unchanged. This means that the active adsorption sites and functional groups in the solution increase with the increase in *m*/*v* value, while the amount of Sb(III) in the solution remains unchanged. With the increase in *m/v* value, more active adsorption sites and functional groups increase the contact and reaction probability with Sb(III) in the solution [53]. This leads to an increase in the adsorption and removal efficiency of Fe_3_-AL_2_CS_2_, while the equilibrium adsorption capacity of Sb(III) decreases, as observed in the study by Zhuang et al. [54]. When the *m*/*v* value is 1.0 g/L, the removal efficiency of Fe_3_-AL_2_CS_2_ for Sb(III) adsorption is 95.07%. At a higher proportion, the *R_e_* value is almost the same, but the *Q_e_* value is 13.09 mg/g. Therefore, the optimal *m*/*v* value for Fe_3_-AL_2_CS_2_ to adsorb Sb(III) is 1.0 g/L.

#### 2.3.3. Effect of Initial Sb(III) Concentration

**Figure 9:**
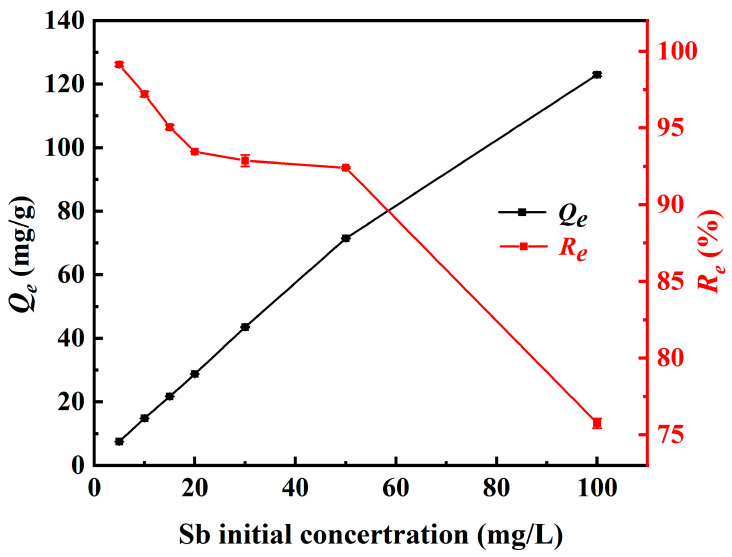
Changes in Sb(III) adsorption capacities (*Q_e_*) and removal efficiency (*R_e_*) with the initial concentration of Sb(III).

The changes in the adsorption performance of Fe_3_-AL_2_CS_2_ for Sb(III) under the initial Sb(III) concentration gradient (*C*_0_ = 5–100 mg/L) are shown in Figure 9. It can be seen from the figure that with the gradual increase in the initial Sb(III) concentration *C*_0_, the removal efficiency (*R_e_*) and equilibrium adsorption capacity (*Q_e_*) of the material show a significant and differentiated changing trend. When *C*_0_ is 5.00 to 100.00 mg/L, the *R_e_* values gradually decrease from 99.13% to 75.75%, while *Q_e_* rapidly increases from 7.51 mg/g to 122.98 mg/g. The high *R_e_* value at this stage can be attributed to the fact that the Fe_3_-AL_2_CS_2_ surface has a high level of active adsorption sites relative to the amount of Sb(III) in the solution. This allows the majority of Sb(III) to be effectively adsorbed. Although the *R_e_* values rapidly decrease from 92.41% to 75.75% when the *C*_0_ is increased from 50 mg/L to 100 mg/L, the *Q_e_* values increase from 71.45 mg/g to 122.98 mg/g. Although the equilibrium adsorption is constantly increasing, the *R_e_* value is gradually decreasing. This may be because the dosage of Fe_3_-AL_2_CS_2_ is fixed, which means that the number of its active adsorption sites and functional groups is also fixed [53]. As the amount of Sb(III) in the solution increases with the increase in the initial concentration, these adsorption sites rapidly become saturated, leaving a large amount of unabsorbed Sb(III) in the solution. This situation leads to a high adsorption equilibrium concentration, but *R_e_* is significantly reduced.

#### 2.3.4. Effect of Adsorption Time

**Figure 10:**
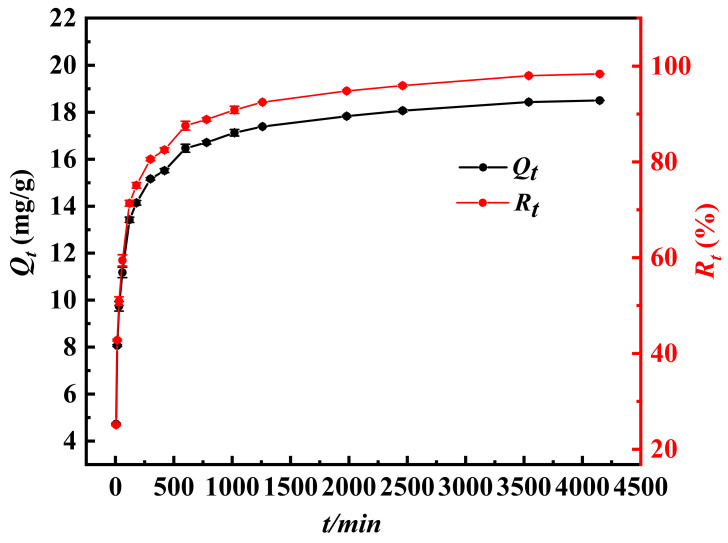
Effect of reaction time on the adsorption of Sb(III) by Fe_3_-AL_2_CS_2._

Figure 10 clearly presents the effect law of adsorption duration on the removal effect of Sb(III). The removal efficiency (*R_e_*) and adsorption capacity (*Q_e_*) of Sb(III) will continue to increase with the increase in adsorption time, but the growth rate of *R_e_* and *Q_e_* slows down after 4148 min. This phenomenon indicates that the equilibrium time of Fe_3_-AL_2_CS_2_ adsorption of Sb(III) is approximately 3540 min. During the entire adsorption process, within the first 5 to 600 min, both the *R_e_* and *Q_e_* of Sb(III) show a gradually increasing trend. At this stage, the adsorption rate is relatively fast. The core reason lies in the fact that there are a large number of unsaturated adsorption sites that have not been occupied and are distributed on the surface of the composite material. Moreover, the concentration of Sb(III) in the solution at the initial stage of adsorption is at a relatively high level. During the period from 780 to 3540 min, the adsorption *R_e_* of Fe_3_-AL_2_CS_2_ for Sb(III) increased from 88.88% to 98.02%. This data further confirmed that the equilibrium duration of its adsorption of Sb(III) was approximately 3540 min. However, compared with the previous period, At this stage, the growth rates of both *R_e_* and *Q_e_* have significantly slowed down compared to the previous period. The specific reason is that most of the adsorption sites of Fe_3_-AL_2_CS_2_ have approached saturation in the initial stage, and the concentration of Sb (III) in the solution has also decreased accordingly [55]. This decrease in concentration will reduce the possibility of Fe_3_-AL_2_CS_2_ and Sb(III) coming into contact and reacting at the adsorption site, ultimately leading to a decrease in the adsorption rate. When the time exceeded 3540 min, although the *R_e_* of Sb(III) slightly increased from 98.02% to 98.39%, the growth rate became slower. This is most likely because most of the active adsorption sites on the surface of Fe_3_-AL_2_CS_2_ were already in a saturated state. Meanwhile, the concentration of Sb(III) in the solution is relatively low, making it difficult for the material to effectively capture Sb (III) in the solution. Based on the above analysis, the adsorption equilibrium duration of Fe_3_-AL_2_CS_2_ for Sb(III) is approximately 3540 min, and the equilibrium adsorption rate exceeds 98%.

#### 2.3.5. Effect of Coexisting Ions

**Figure 11:**
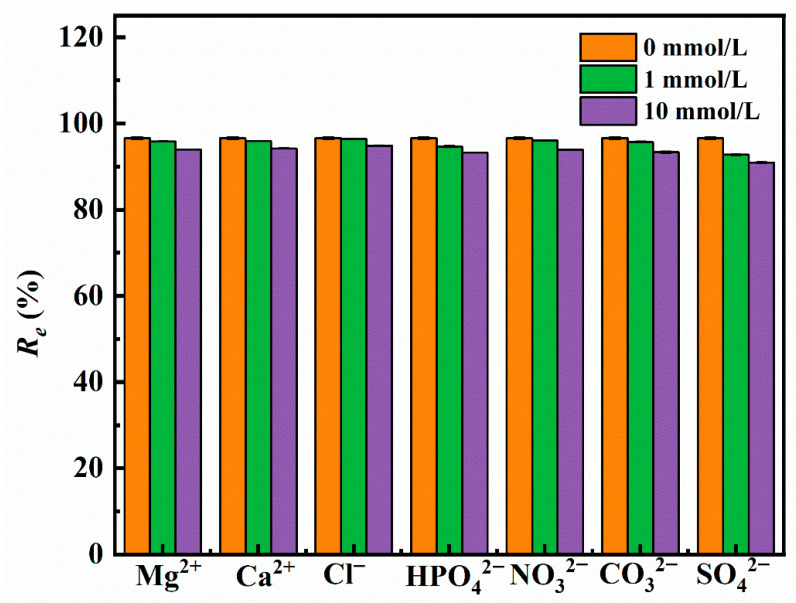
The influence of coexisting ions on the removal rate *R_e_* of Sb(III).

To study the effects of common anions (NO_3_^−^, SO_4_^2−^, Cl^−^, HPO_4_^2−^, CO_3_^2−^) and cations (Ca^2+^, Mg^2+^) in water on the adsorption of Sb(III) by Fe_3_-AL_2_CS_2_, the above ions are added to the Sb(III) solution at concentrations of 1.0 mmol/L and 10.0 mmol/L, respectively. Adsorption performance tests are conducted, and the results are shown in Figure 11. The adsorption results are compared with those without coexisting ions (*R_e_* = 96.59%). At concentrations of 1.0 mmol/L and 10.0 mmol/L, Ca^2+^, Mg^2+^, NO_3_^−^, and Cl^−^ have less effect on the Fe_3_-AL_2_CS_2_ adsorption of Sb(III), which is consistent with previous reports [56]. This may be attributed to the weak binding of Cl^−^ reaching the surface of the adsorbent through the outer sphere complex [57]. However, SO_4_^2−^ demonstrates the strongest inhibitory effect, with *R_e_* values reduced by 3.84% and 5.67%, respectively. This is attributed to the stronger binding property of FeO(OH) to SO_4_^2−^ at higher concentrations and lower pH conditions. Secondly, there are HPO_4_^2−^ and CO_3_^2−^. Under the concentration condition of 1 mmol/L, the *R*_e_ values decrease by 1.94% and 0.9%, respectively. Under the condition of 10 mmol/L, the *R_e_* values decrease by 3.33% and 3.28%, respectively. This is attributed to the fact that HPO_4_^2−^ and CO_3_^2−^ reduce the adsorption effect by forming spherical complexes with heavy metal ions and competing with Sb(III) for the active sites on the adsorbent. Therefore, the adsorption reaction is significantly affected, which is consistent with previous research [17]. By comparing with previous relevant studies, it can be seen that the influence of the above-mentioned common ions on the adsorption of Sb(III) by Fe_3_-AL_2_CS_2_ is significantly lower than that reported in the existing literature [58,59].

#### 2.3.6. Regeneration Experiment

**Figure 12:**
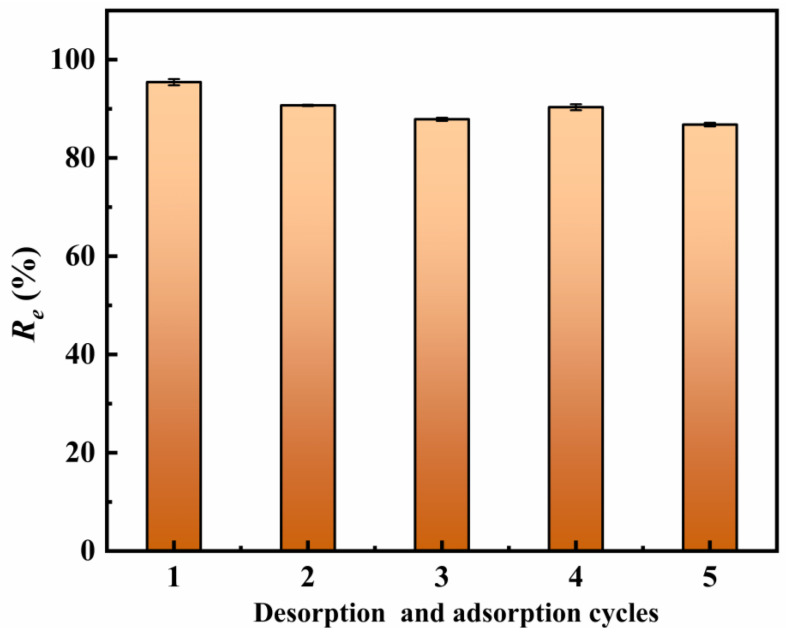
Changes in the removal efficiency (*R_e_*) of Fe_3_-AL_2_CS_2_ over five times of regeneration cycles.

The regeneration and reusability of adsorbents are important factors for materials’ practical application [60]. Figure 12 shows the removal efficiency (*R*_e_) of Sb(III) after desorption using 0.1 mol/L NaOH. The *R_e_* value of Sb(III) by Fe_3_-AL_2_CS_2_ in the first regeneration could reach 96.59%. After five regenerations, although the *R_e_* values of Sb(III) by Fe_3_-AL_2_CS_2_ decrease, they remain at 86.8% in the end. This indicates that the Fe_3_-AL_2_CS_2_ in this study has good reusability.

### 2.4. Adsorption Characteristics

#### 2.4.1. Adsorption Kinetics

**Figure 13:**
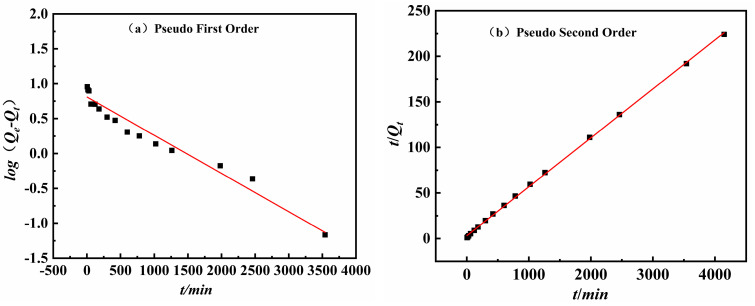
Fitting of the adsorption kinetics model of Sb(III) Fe_3_-AL_2_C_2_ adsorption: (**a**) pseudo-first-order kinetics model; (**b**) pseudo-second-order dynamic model; isothermal adsorption model fitting.

To study the adsorption rate and behavior of Sb(III) on Fe_3_-AL_2_CS_2_ during the adsorption process, pseudo-first-order and pseudo-second-order kinetic models are fitted using experimental data. The fitting results of each adsorption kinetics model are shown in Figure 13a,b, and the relevant parameters are listed in Table 1. The results show that the pseudo-second-order coefficient of determination (*R*^2^) is 0.99, and the fitted *Q_e_* value of 18.62 mg/g is closer to the experimental result of 31.37 mg/g. *R*^2^ obtained by the pseudo-first-order model is 0.93. This means that the pseudo-second-order kinetic model fits the adsorption process of Sb(III) on Fe_3_-AL_2_CS_2_ better than the pseudo-first-order kinetic model, indicating that Sb(III) adsorption is mainly chemical.

#### 2.4.2. Isothermal Adsorption

**Figure 14:**
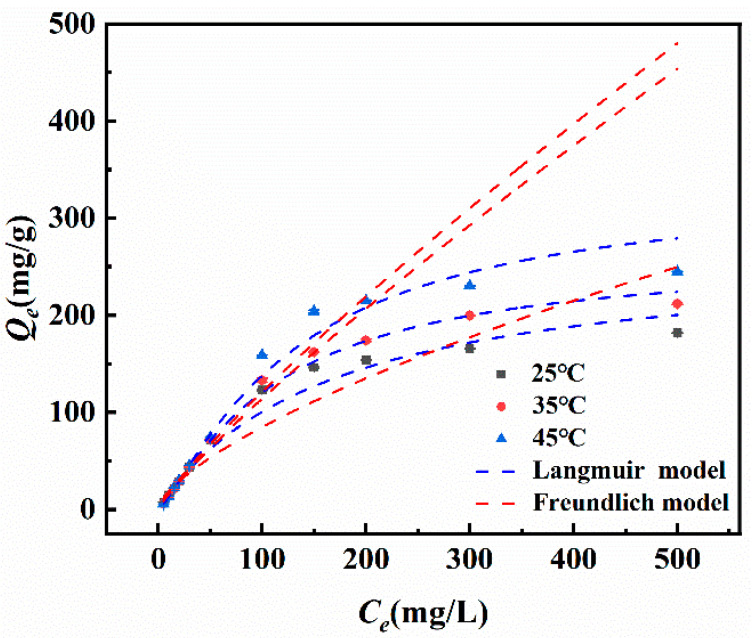
Adsorption isotherm fitting curves of Sb(III) on the Fe_3_-AL_2_CS_2_ composite.

Figure 14 shows the experimental results of isothermal adsorption of Fe_3_-AL_2_CS_2_ by Sb(III) at 25 °C, 35 °C, and 45 °C, as well as the fitting curves of the Langmuir and the Freundlich models. The relevant parameters are listed in Table 2. It has been observed that the equilibrium adsorption capacity (*Q_e_*) increases with the temperature rise, indicating that the adsorption capacity of Fe_3_-AL_2_CS_2_ for Sb(III) enhances with the increase in temperature. The fitting results show that the Freundlich model fits poorly at different temperatures, and the *K*_F_ and 1/*n* values are 3.84 and 0.67, respectively (At 25 °C). The 1/*n* value is between 0 and 1, and the coefficient of determination (*R*^2^) is 0.95, indicating that it is advantageous to adsorb Sb(III). In contrast, the Langmuir model demonstrates better fitting, with a maximum adsorption capacity (*Q_m_*) of 266.58 mg/g for Sb(III) at 25 °C and *R*^2^ of 0.99, indicating that the adsorption of Sb(III) by Fe_3_-AL_2_CS_2_ occurs at the functional groups/binding sites on the surface of the adsorbent, which is regarded as monolayer adsorption.

### 2.5. Adsorption Mechanism

**Figure 15:**
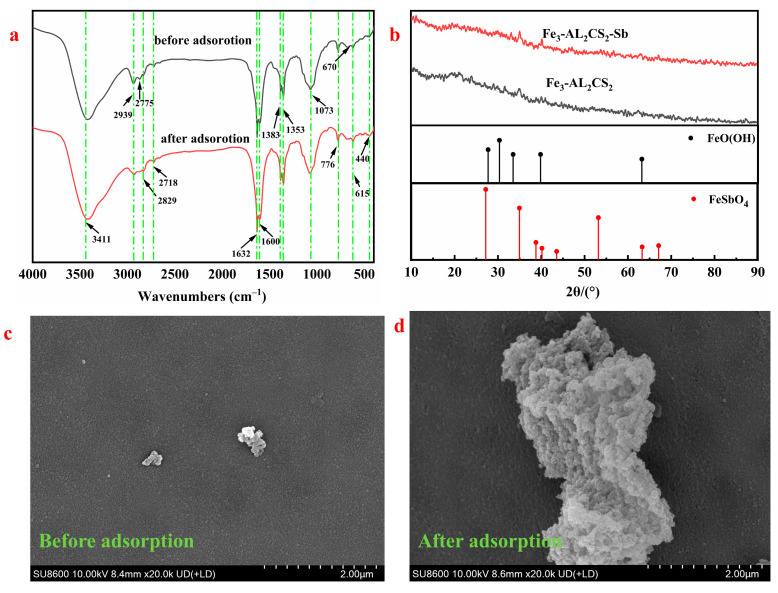
Comparison of FTIR (**a**), XRD (**b**), and SEM (**c**,**d**) of Fe3-AL2CS2 before and after adsorbing Sb(III).

Figure 15a shows the FTIR spectra of Fe_3_-AL_2_CS_2_ before and after adsorbing Sb(III). The characteristic peak at 3411 cm^−1^ is the O–H tensile vibration peak, indicating the presence of hydroxyl groups in the material. The peak is enhanced after adsorption at 3411 cm^−1^, which may be due to the formation of hydroxyl complexes between Sb(III) and –OH [10,61]. This indicates that the adsorption process of Sb(III) is related to O–H. The 1383–1353 cm^−1^ peak is attributed to the C=C vibration, and the 1073 cm^−1^ peak corresponds to the C–O–C stretching vibration. Both bands change after absorbing Sb(III), which is related to the electron transfer caused by Sb(III) coordination. This is attributed to the transfer of oxygen atom electrons to the empty Sb(III) orbital. The electronic states of the C=C and C–O–C bonds have been changed [53]. This indicates that these functional groups are involved in the adsorption mechanism. Consistent with the research of Simic et al. [62], changes in the C–O and C=O bands are observed during the adsorption process of metal ions, suggesting the existence of chemical interactions, such as ligand exchange and chemical adsorption. In addition, a new peak appears at 440 cm^−1^ after adsorption. This may be due to the Fe–O vibration band moving to a lower wavelength, indicating the formation of a new complex (e.g., Sb–O–Fe). This indicates that Fe–O is involved in the adsorption process of Sb(III).

After adsorbing Sb(III), the XRD patterns of the Fe_3_-AL_2_CS_2_ composite material show key characteristic changes, as shown in Figure 15b. The new peaks appearing at 2*θ* = 33.1° and 40.1° after adsorption are consistent with the XRD standard characteristic peaks of FeSbO_4_. This indicates that Sb(III) has been successfully adsorbed onto the surface of the composite material and may form the FeSbO_4_ compound. The formation of this mineral phase is key evidence for the removal and stabilization of Sb(III) from the solution to the solid phase. In addition, the intensity of the characteristic peak of FeO(OH) increases at 2*θ* = 35°, supporting the positive role of the FeO(OH) group in the adsorption process of Sb(III) [63]. This enhancement indicates that the FeO(OH) functional group is related to the removal of Sb(III). The enhancement of FeSbO_4_ and FeO(OH) peaks indicates that complex chemical reactions occur simultaneously on the material surface, thus facilitating the effective removal and stabilization of Sb(III).

As shown in the SEM image (Figure 15c) before adsorption, the particles show excellent dispersion, existing in the form of nanoscale discrete particles with relatively regular shapes, smooth and dense surfaces, and no obvious agglomeration phenomenon. The surface is rough and porous, with the boundaries of the original discrete particles blurred, forming an interwoven structure of flocculent and spongy. After adsorbing Sb(III) (Figure 15d), the particles agglomerate into large-sized aggregates, with a rough and porous surface, forming an interwoven structure of flocculent and spongy. The EDS spectra before and after adsorption are shown in Table 3. Before Sb(III) adsorption, the contents of elements in the Fe_3_-AL_2_CS_2_ composite material are C (46.74%), O (38.99%), Fe (31.69%), and N (2.58%). After the Fe_3_-AL_2_CS_2_ complex adsorbs Sb(III), the contents of C and N decrease by 4.77% and 1.55%, respectively, while the content of Fe significantly increases to 52.58%, the content of O significantly decreases to 19.47%, and Sb is 5.25%. These results indicate that the Fe–O group in Fe_3_-AL_2_CS_2_ plays a key role in the adsorption process of Sb(III) and is the main adsorption site of Sb, which is consistent with the analyses of XRD and FTIR.

### 2.6. Performance Analysis

As shown in Table 4, the maximum adsorption capacity of Fe-ALCS for Sb(III) reaches 266.58 mg/g, which is significantly superior to other modified adsorption materials. The adsorption capacity of rGO aerogel for Sb(III) is 168.59 mg/g [64], FeBC is 64.0 mg/g (the adsorption capacity increases by seven times after adding persulfate) [65], and NMCS is 69.85 mg/g [66]. FeGB-gel was 113.1 mg/g [67], CH_1_BC was 168 mg/g, and biochar was loaded with magnesium ferrite to synergistically remove Sb(III) [43]. The highly efficient Sb(III) removal performance of Fe-ALCS provides high-quality materials and technical support for the treatment of antimony-containing wastewater.

### 2.7. Performance of Aerogel in Treating Simulated Multi-Metal Wastewater

**Figure 16:**
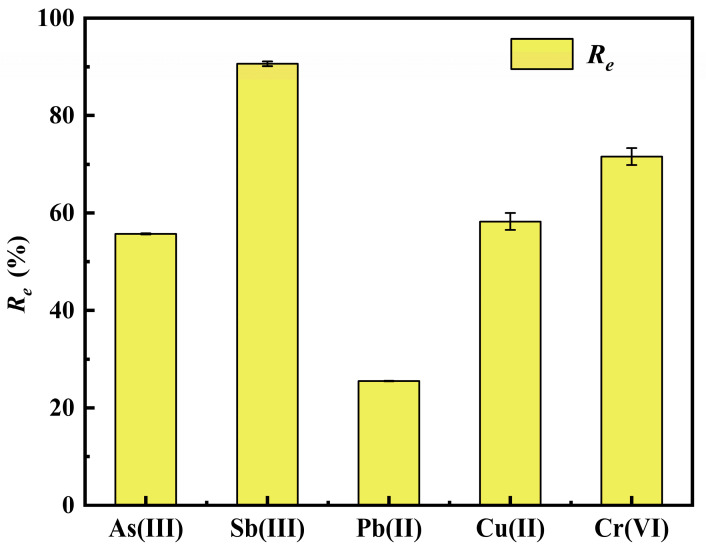
The removal effect of aerogels on multi-metal wastewater.

To evaluate the actual wastewater treatment effect of the prepared Fe-ALCS aerogel (i.e., the iron-modified alkali lignin chitosan aerogel synthesized by freeze-drying), and to verify its applicability in the remediation of actual polymetallic contaminated wastewater, this study conducted batch experiments using a simulated solution containing multiple heavy metals. The initial concentrations of each heavy metal in the simulated solution are as follows: As(III) 14.17 mg/L, Sb(III) 13.43 mg/L, Pb(II) 7.01 mg/L, Cu(II) 10.30 mg/L, and Cr(VI) 8.78 mg/L. The experimental results are shown in Figure 16. This adsorbent exhibits targeted and highly efficient adsorption performance for Sb(III), with a removal rate (*R_e_*) of over 90%, which is consistent with its designed function for Sb(III) removal. Meanwhile, the adsorbent also has a certain adsorption effect on other coexisting heavy metals. Among them, the removal rates of As(III), Cr(VI), and Cu(II) are 55%, 71%, and 58%, respectively. In contrast, its adsorption effect on Pb(II) is relatively weak, with a removal rate of only 25%. In conclusion, this performance analysis confirms that Fe-ALCS aerogel, as an environmentally friendly and renewable biomass adsorbent, has the potential to simultaneously remove Sb(III) and coexisting metals such as As(III), Cr(VI), and Cu(II) from actual wastewater.

## 3. Materials and Methods

### 3.1. Materials

Alkaline lignin (AL, (C_30_H_25_ClN_6_)) is purchased from Shanghai Baishun Biotechnology Development Co., Ltd. (Shanghai, China). The product is a brownish-brown powder, with a molecular weight of 505.01. Chitosan (CS, (C_6_H_11_O_4_)_n_) was purchased from Lanjing Technology Development Co., Ltd. (Shanghai, China). It is in the form of white powder, with a deacetylation degree of 90.45% and a molecular weight of 70–80 × 10^4^ Da. Glutaraldehyde (50%) is purchased from Aladdin Reagent Co., Ltd. (Shanghai, China). Potassium antimonyl tartrate trihydrate (C_8_H_4_K_2_O_12_Sb_2_∙3H_2_O) is the source of Sb(III) and is purchased from Macklin Biochemical Co., Ltd. (Shanghai, China). Other AR grade reagents and chemicals, including HCl, FeCl_2_⋅4H_2_O, NaOH, anhydrous ethanol, acetic acid, and methanol, are all purchased from Xilong Science Co., Ltd. (Shanghai, China). During the entire research process, deionized water (18.2 MΩ∙cm) is prepared using the Milli-Q water system (Millipore, Burlington, MA, USA).

### 3.2. Preparation of Fe-ALCS Composite Materials

**Figure 17:**
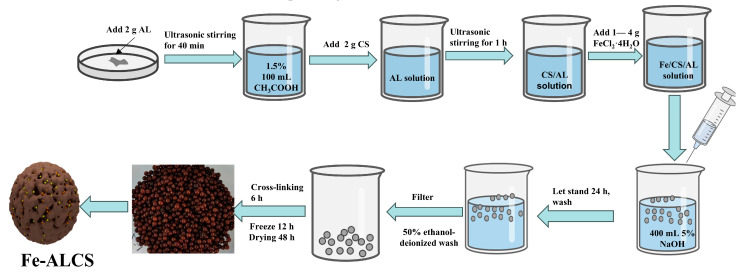
Schematic diagram of the synthesis process of Fe-modified basic lignin chitosan aerogel microspheres (Fe-ALCS).

The synthesis process of the composite material in this study is presented in Figure 17. The preparation process of Fe-ALCS composite materials is as follows:

(1) Add 2 g of alkaline lignin (AL) powder to 100 mL of 1.5% acetic acid solution. Then, the mixture is put into a beaker, and ultrasonic treatment and stirring are carried out for 40 min at the same time, so that the AL is evenly dispersed. Subsequently, 2 g of powder chitosan (CS) is added, and after ultrasonic stirring for about 1 h, the chitosan is basically dissolved. 

(2) After that, manually add FeCl_2_·4H_2_O of different masses to the ALCS mixture. Fe, AL, and CS are mixed according to the mass ratios of 1:1:1, 2:1:1, 3:1:1, and 4:1:1. The mixture is continuously stirred until it is evenly dispersed to obtain an Fe/AL/CS mixture solution.

(3) Then, the obtained Fe/AL/CS mixture solution is dropped into 400 mL of 5% NaOH solution to form aerogel microspheres. After being placed in the dark at room temperature for 24 h, the aerogel microspheres are filtered from the NaOH solution, and they are washed until the washing solution is nearly neutral.

(4) The obtained aerogel microspheres are then placed in 200 mL of a 5% glutaraldehyde mixed solution. These materials are cross-linked under oscillation in a room temperature water bath (180 rpm) for 6 h and then repeatedly washed with deionized water until neutral. Then, the aerogel microspheres are placed in a freeze dryer (Sihuan Qihang LGJ-12A model, Sihuan Qihang Technology Co., Ltd., Beijing, China), frozen for 12 h, and finally dried for 48 h. The final four brown composite microspheres with a diameter of approximately 3 mm and a mass of 5.0 ± 0.1 mg are named Fe_1_-AL_2_CS_2_, Fe_2_-AL_2_CS_2_, Fe_3_-AL_2_CS_2_, and Fe_4_-AL_2_CS_2_ (note: the numbers in the subscript Fe_1_-AL_2_CS_2_ are presented as mass ratios), respectively.

### 3.3. Batch Adsorption Experiment

First, accurately weigh 50 mg of Fe-ALCS composite microspheres and carefully transfer them into a clean centrifuge tube of 100 mL specification to ensure that the microspheres do not adhere to the tube wall. Subsequently, the prepared 50 mL 10 mg/L Sb(III) ions solution was injected into the tube. 0.1 mol/L HCl solution and 0.1 mol/L NaOH solution were used as pH regulators. Through multiple small drops and real-time monitoring with a pH meter, finally stabilize the pH of the system within the set range of 3.0 ± 0.1. After the system construction is completed, the centrifuge tubes are covered and sealed and then transferred to a constant temperature water bath shaker. The shaker parameters are set at 25 °C temperature and 180 rpm oscillation frequency. Under these conditions, the adsorption reaction is carried out, and the oscillation is continuous for 48 h to ensure that the adsorption process reaches a thermodynamic equilibrium state. After the adsorption equilibrium, remove the centrifuge tube and let it stand for a moment. Once the microspheres inside the tube naturally settle, use a 10 mL disposable sterile syringe to slowly draw 9 mL of the upper clear liquid. Immediately filter the drawn clear liquid through a 0.45 μm aqueous microporous filter membrane that has been rinsed with ultrapure water. Remove any possible residual microsphere particles and impurities to prevent them from interfering with the subsequent detection signals. After the filtrate collection was completed, an inductively coupled plasma optical emission spectrometer (ICP-OES, model Optima 7000DV by PerkinElmer Instruments, Inc.; the production site is Waltham, MA, USA) was used. The concentration of Sb(III) is determined on it. The adsorption equilibrium concentration (*C_e_*), removal efficiency (*R_e_*), and adsorption equilibrium capacity (*Q_e_*), respectively, are determined and calculated according to Equations (1) and (2).

(1) $$R_{e}=\frac{C_{0}-C_{e}}{C_{0}}\times 100\%$$

(2) $$Q_{e}=\frac{C_{0}-C_{e}}{m}\times v$$

where *R_e_* represents the removal efficiency of Sb(III) at equilibrium, %; *C*_0_ is the initial Sb(III) concentration, mg/L; *C_e_* is the Sb(III) concentration at equilibrium, mg/L; *Q_e_* is the adsorption capacity of Sb(III) at equilibrium, mg/g; *v* is the volume of the solution containing Sb(III), L; and *m* represents the mass of the adsorbent, g.

### 3.4. Isothermal Adsorption Experiment

Take 50 mg of Fe-ALCS composite microspheres and place them into a 100 mL centrifuge tube. Add 50 mL of Sb(III) solution (with concentrations of 5, 10, 15, 20, 30, 50, 100, 150, 200, 300, 500 mg/L, respectively). Adjust the pH of the system to 3, place it in a constant temperature water bath shaker at 25 °C for incubation for 48 h, filter with a 0.45 μm filter membrane, and determine the concentration of Sb(III) in the filtrate.

The experimental results are used to fit the Langmuir and the Freundlich isotherm models as follows:

(3) $${Langmuir:Q}_{e}=\frac{Q_{m}K_{L}C_{e}}{1+K_{L}C_{e}}$$

(4) $$Freundlich:Q_{e}=K_{F}C_{e}^{1/n}$$

where *Q_e_* represents the adsorption capacity at equilibrium, mg/g; *C_e_* is the concentration of Sb(III) at equilibrium, mg/L; *Q_m_* is the maximum adsorption capacity of the material for Sb(III), mg/g; and *K_L_* is the Langmuir equilibrium constant related to the strength of the adsorption interaction. *K_F_* and 1/*n* are the Freundlich equation constants for adsorption equilibrium and strength, respectively.

### 3.5. Adsorption Kinetics Experiment

Place 50 mg of Fe-ALCS composite microspheres in a 100 mL centrifuge tube and add 50 mL of 20 mg/L Sb(III) solution. Adjust the pH value to 3.0 and collect samples at intervals of 5, 15, 30, 60, 120, 180, 300, 420, 600, 780, 1020, 1260, 1980, 2460, 3540, and 4148 min. At regular intervals, the solution is transferred from a single test tube at the corresponding time points, filtered through a 0.45 μm filter, and the concentration of Sb (III) is measured.

To evaluate the kinetic adsorption mechanism of Sb(III) on the adsorbent, the pseudo-first-order kinetics shown in Equation (5) and the pseudo-second-order kinetics shown in Equation (6) are applied to simulate the adsorption kinetics of Sb(III) on the adsorbent. The pseudo-first-order kinetic model is based on the membrane diffusion theory and assumes that the adsorption process is controlled by physical adsorption [68]. The pseudo-second-order kinetic model assumes that the adsorption process involves the sharing or transfer of electron pairs between the adsorbent and the adsorbate, and it is determined by chemical adsorption [68].

(5) $$\log(Q_{e}-Q_{t})=\log Q_{e}-\frac{k_{1}}{2.303}t$$

(6) $$\frac{t}{Q_{t}}=\frac{1}{K_{2}Q_{e}^{2}}+\frac{t}{Q_{e}}$$

where *Q_e_* (mg/g) and *Q_t_* (mg/g) represent the adsorption capacities at equilibrium for Sb(III) and reaction time (*t*), respectively. *K*_1_ and *K*_2_ are the first-order and second-order rate constants, respectively.

### 3.6. Analytical Techniques

The concentration of Sb(III) in aqueous solution is determined by an inductively coupled plasma optical emission spectrometer (ICP-OES, Optima 7000DV, PerkinElmer Instruments, Inc., Waltham, MA, USA). The surface morphology and elemental analysis of Fe-ALCS are determined by JSM-7900F SEM-EDS (JEOL, Tokyo, Japan). The ASAP 2460 physical adsorption analyzer (Micromeritics Instrument Corporation, Norcross, GA, USA) is used for BET testing of Fe-ALCS. The Model 8604 Vibrating Sample Magnetometer (VSM) (manufactured by Lake Shore, Westerville, OH, USA) is used to characterize the magnetic properties of Fe-ALCS. The IS10 FTIR spectrometer (Thermo Fisher, Waltham, MA, USA) is used to determine the functional groups of Fe-ALCS. The crystal structure of Fe-ALCS is determined by X’Pert3 powder multifunctional XRD (Panaco, London, UK, copper target, λ = 1.54056 A). The scanning step size, speed, and range are 0.02626°, 0.6565°/s, and 5°–90° (2*θ*), respectively.

## 4. Conclusions

In this study, by taking metal Fe, basic lignin, and chitosan as raw materials, four aerogel composite materials (Fe_1_-AL_2_CS_2_, Fe_2_-AL_2_CS_2_, Fe_3_-AL_2_CS_2_, and Fe_4_-AL_2_CS_2_) with different mass ratios are prepared. The research found that Fe_3_-AL_2_CS_2_ not only has the best adsorption performance for Sb(III) but also has better spheroidization properties than Fe_4_-AL_2_CS_2_. Therefore, Fe_3_-AL_2_CS_2_ was selected for the subsequent experiments. The adsorption capacity of Fe_3_-AL_2_CS_2_ for Sb(III) increased rapidly from 7.51 mg/g to 122.98 mg/g with the increase in Sb(III) concentration, but the removal efficiency gradually decreased from 99.13% to 75.75%. Regarding the mass-to-volume ratio (*m*/*v*), as the *m*/*v* value increases, the adsorption capacity gradually decreases from 76.50 mg/g to 12.79 mg/g, while the removal efficiency gradually increases, from 80.48% to 97.20%. The adsorption performance of this material is better under acidic conditions. As the pH value increases from 3 to 11, the adsorption capacity decreases from 18.47 mg/g to 17.67 mg/g. Other optimal removal conditions for Sb(III) are pH = 3, *m*/*v* = 1.0, and initial concentration (*C*_0_ = 20 mg/L). And, the adsorption equilibrium time is 3540 min for Sb(III) adsorption by Fe_3_-AL_2_CS_2_. Coexisting ions, including anions NO_3_^−^, SO_4_^2−^, Cl^−^, HPO_4_^2−^, and CO_3_^2−^ and cations of Ca^2+^ and Mg^2+^, have little effect on Sb(III) adsorption. The adsorption of Sb(III) by Fe_3_-AL_2_CS_2_ conforms to the pseudo-second-order kinetic model (*R*^2^ = 0.99), with chemical adsorption playing a dominant role. The Langmuir model fits well, with a maximum *Q_e_* of 266.58 mg/g, indicating monolayer adsorption. Characterization analysis of BET, XRD, FTIR, and SEM-EDS confirms that Fe_3_-AL_2_CS_2_ has a porous microspherical structure, and FeO(OH) is the main adsorption site of Sb(III). Functional groups such as C–OH and C–O in the ALCS matrix assist in adsorption, reducing Fe dissolution. Eventually, efficient adsorption for Sb(III) is achieved through the synergy of ligand exchange and complexation.

## Figures and Tables

**Table 1:** Adsorption kinetics model parameters.

Temperature (°C)	Pseudo-First-Order			Pseudo-Second-Order		
	*Q_e_* (mg/g)	*K*_1_	*R* ^2^	*Q_e_* (mg/g)	*K*_2_	*R* ^2^
25	2.25	−5.48	93	18.62	0.05	99

**Table 2:** The Langmuir and the Freundlich fitting data table.

Temperature (°C)	Langmuir			Freundlich		
	*Qe* (mg/g)	*K_L_*	*R* ^2^	*K_F_*	1/*n*	*R* ^2^
25	266.58	0.006	0.99	3.84	0.67	0.94
35	262.39	0.003	0.99	2.18	0.85	0.95
45	334.24	0.002	0.99	2.34	0.86	0.93

**Table 3:** The element compositions of solid samples before and after adsorption.

Elements	C	N	O	Fe	Sb
Before adsorption (%)	26.74	2.58	38.99	31.69	-
After adsorption (%)	21.97	1.03	19.47	52.58	5.25

**Table 4:** Comparison of the adsorption capacity of Sb(III) by various adsorbents.

Adsorbent	Heavy Metal	Adsorption Capacity (mg/g)	References
NMCS	Sb(III)	69.58	[66]
FeGB gel	Sb(III)	113.1	[67]
rGO aerogel	Sb(III)	168.59	[64]
FeBC	Sb(III)	64.0	[65]
CH_1_BC	Sb(III)	168	[43]
Fe-ALCS	Sb(III)	266.58	This study

## Data Availability

No new data were created or analyzed in this study. Data sharing does not apply to this article.
